# Motivations, experiences and aspirations of trainee nursing associates in England: a qualitative study

**DOI:** 10.1186/s12913-020-05676-7

**Published:** 2020-08-26

**Authors:** Rachel King, Tony Ryan, Emily Wood, Angela Tod, Steve Robertson

**Affiliations:** Health Sciences School, Division of Nursing and Midwifery, University of Sheffield, Room 3a B02, Barber House Annexe, 3a Clarkehouse Road, Sheffield, S10 2LA UK

**Keywords:** Trainee nursing associates, Workforce, Focus groups, Role identity

## Abstract

**Background:**

The nursing associate role was developed in England in response to the ‘Shape of Caring’ review. It has been implemented to fulfil two aims; to bridge the gap between registered nurses and healthcare assistants, and to provide an alternative route into registered nursing in light of workforce shortages.

Other high income countries deploy second level nurses within their healthcare systems, however the UK has a turbulent history with such roles. The previous state enrolled nurse was phased out in the 1990s, and more recently the assistant practitioner (AP) role has faced wide variation in titles, scope and pay. Little is known about those who have embarked on the new nursing associate training course and their experiences of the role.

**Methods:**

An exploratory qualitative study was undertaken using focus groups of trainee nursing associates to generate in-depth discussion about their motivations, experiences of training, and career aspirations.

Three focus groups (*n* = 15) took place in December 2018 using a purposive sample of trainee nursing associates registered at a University in the North of England. Two researchers facilitated each group discussion at a time and place convenient for participants. The discussions were audio recorded, transcribed and data was analysed thematically.

**Results:**

This study found that trainee nursing associates are motivated by affordable, local, career development. During training they face challenges relating to clinical support, academic workload and uncertainty about future career opportunities. They experience role ambiguity both individually and across the wider organisation. Trainee nursing associates rely on broad support networks to build their occupational identity.

**Conclusions:**

The barriers and facilitators of trainee nursing associate personal development have implications for policy and practice relating to recruitment and retention. The results increase our understanding of this emerging role, and have informed the development of a larger longitudinal cohort study. Further research is required to evaluate the impact of this new role.

## Background

The nursing associate (NA) role has been introduced in England in response to recommendations set out in the ‘Shape of Caring’ review [1]. The aim of the role is twofold; to bridge the gap between healthcare assistants (HCAs) and registered nurses (RNs), while simultaneously offering an alternative route into nursing. This is particularly important in the current context, as the UK health service is under increasing pressure due to an aging population and workforce shortages [2].

Little is known about the new NA role as the first students only qualified in January 2019, however lessons can be learned from previous research on similar roles such as the state enrolled nurse (SEN) and assistant practitioner (AP).

NA training combines academic and work-based learning, incorporating all four fields of nursing [3]. A service evaluation of 39 trainee nursing associates (TNAs) in the Northeast of England raised a number of important issues related to TNAs, including role clarity, placement models, mentorship and protected learning time [3, 4]. A larger internal evaluation of the TNA pilot programme has recently been commissioned by Health Education England [5]. The current study builds on these previous evaluations, exploring the motivations, experiences and aspirations of trainee nurse associates (TNAs) in one particular Higher Educational Institute (HEI), and makes suggestions for future research on this emerging role.

The healthcare workforce in the UK has experienced a significant shift from traditional role boundaries over recent years. Nurses have taken on more medical tasks and, consequently, HCAs have adopted more advanced skills in patient care [6]. The introduction of NA training has offered HCAs the opportunity of formal career development [1]. Two pilot cohorts of TNAs, with around 1000 trainees each, were funded across 35 sites in England in January 2017 and April 2017 [7], with the first student qualifying in January 2019. Since then an apprenticeship model has been introduced with an additional 5000 trainees recruited in 2018, and plans for a further 7500 by 2020 across England [8].

Globally HCAs and similar support roles have lacked formal training and opportunities for career progression [1, 9, 10]. One US study, found that despite high aspirations to become RNs, few support workers progressed their careers within the healthcare setting [10]. However, career development, further education, and increased wages are all motivators for progression if given the opportunity [10–12].

Nursing workforce shortages of more than 40,000 are a growing concern [13]. There are currently 100,000 vacancies across the NHS in England, with a predicted rise to 350,000 by 2030 [2]. In the UK, applications to study nursing, particularly among mature students, have fallen since the removal of the student bursary and many nurses are leaving the profession due to difficult working conditions, exacerbated by poor staffing levels [14]. There has been a 30% increase in support staff (compared to 10% increase in nurses), since 2013, as organisations attempt to meet increasing patient need [15]. In this context, some have raised concerns that substitution of RNs with less well trained staff is unlikely to provide an effective solution to the nursing workforce crisis [16]. One policy assumption is that 50% of NAs will go on to become RNs, with the transition to RN being smoother and with lower attrition rates than other student nurses [1, 7, 17].

Several other high income countries such as the USA, Australia and New Zealand deploy second level nurses with varying titles such as enrolled nurses, licenced vocational nurses, or licenced practical nurses [18]. In the UK, the new NA role has been compared to both state enrolled nurses (SENs) and assistant practitioners (APs) [19, 20]. The SEN role was phased out in the 1990s after the restructuring of nurse education following criticism of its lack of career progression and the limitations imposed by employers in narrow interpretations of role competencies [19, 20]. More recently the assistant practitioner (AP) role was introduced [6], however it has experienced wide variation in titles, scope of practice and pay due to a lack of national regulation [21, 22].

TNAs gain a Foundation Degree over 2 years and, unlike APs, are required to register with the NMC, gain clinical exposure and experience in all fields of nursing, and adhere to the standards of proficiency for nursing associates [23]. Griffiths and Robinson [24] argue that national regulation of such bridging roles would improve patient safety by ensuring mandatory, standardised training, controlling access to employment, and clarifying the scope of practice.

New roles in healthcare are associated with widespread ambiguity [9]. One mechanism by which healthcare workers can support each other is through a community of practice. These occur either formally or informally, with the common aims of improving practice and exchanging knowledge. Characteristics of communities of practice include social interaction, knowledge sharing, knowledge creation, and identity building [25]. Naturally occurring communities of practice can be exploited by formally developing them for the purpose of translating knowledge into action [26]. The current study contributes to the emerging evidence base on an under-researched area of workforce development.

## Methods

### Aim

The aims of this study were to explore TNA motivations, experiences, and career aspirations, and to generate data that will inform future research, including the development of a larger longitudinal cohort study.

### Design

This exploratory study used qualitative research approaches including focus group discussions with TNAs and thematic analysis techniques [27]. Focus groups are commonly used in exploratory research [28], and are particularly useful in generating data through group interaction which progresses back and forwards, producing deeper levels of response than can be gained from individual interviews [29]. The questions used to guide the focus group discussions are presented in Table 1.

**Table 1:** Topic guide

**Motivations for undertaking TNA training**	
What motivated you to undertake NA training? Where has everyone worked prior to starting NA training and in what role? Can you talk about the reasons for applying for NA training? Why not stay in your previous role? What are the benefits of being an NA?	
**Experiences of NA training**	
What’s it like being a TNA? • What were your expectations? • Has the TNA role met your expectations? • What do you like best about the new role? • What do you think some of the problems are? • How does being a TNA differ from your previous roles? • How does mentoring/supervision work? What are your experiences of learning in practice? • How do other healthcare professionals respond to your new role? • Where do you view the TNA role in the wider health care team? • What sorts of factors influence your confidence in the role	
**Aspirations for future development/ transition to registered nurse**	
What are your thoughts about what you’ll do after NA training? • Do you see this as a route into nursing? • What are the benefits of this route into nursing?	
**If you could say one thing you’ve come away with from the training what would it be?**	
**Is there anything else you would like to say about your experiences of being a TNA?**	

### Sampling

Purposive sampling was used to select TNA participants. The sampling frame included all TNAs registered on two cohorts at a University in the North of England. The first group were from the April 2017 pilot cohort and the second group were from the June 2018 apprenticeship cohort. Sampling aimed to achieve maximum diversity in terms of age, gender, and previous health care work experience. Table 2 shows that this was only partially achieved and that the groups were very homogenous in terms of ethnicity, relatively homogenous in terms of prior working background, but more heterogeneous in relation to gender and age.

**Table 2:** Demographics

	Focus group 1	Focus group 2	Focus group 3	Total
**Number of participants**	3	3	9	15
**Male**	3	1	2	6
**Female**	0	2	7	9
**Age 21–30**	2	2	4	8
**Age 31–40**		1	4	5
**Age 41–50**	1		1	2
**Ethnicity- White British**	3	3	9	15
**Background in adult healthcare**	0	3	7	10
**Background in mental health care**	2	0	2	4
**Background in learning disability care**	1	0	0	1

### Recruitment

All TNAs on the two programmes were sent information sheets via email and those interested in taking part were requested to contact the research team directly. A £10 gift voucher was offered to each participant, not as a payment incentive to participate but as a small token of recognition for the time they had given as is common in qualitative studies. Fifteen TNAs agreed to take part in the study out of a total of 70 students. Three focus groups were undertaken, one from the first cohort and two from the second cohort (to ensure convenience for students at different sites). In line with suggested guidelines for qualitative research, the numbers in studies focusing on experiences should be big enough to demonstrate patterns across a data set but small enough to retain a focus on individual experiences ([30] p.45). We believe a group of 15 TNA’s provides this balance and data analysis suggests the same in terms of reaching saturation of themes. These same guidelines recommend between 2 and 4 focus groups for studies using a thematic analysis design ([30] p.50).

### Data collection

Focus groups took place in December 2018, at times and academic settings convenient for participants. They were led by two facilitators, one male and one female, using a topic guide (see Table 1). The topic guide covered TNAs’ reasons for applying to the course, experiences of the role (whether it met expectations, differences compared to previous roles, learning in practice), and future career aspirations. The topic guide was piloted with a TNA lecturer and a TNA.

Three focus groups were undertaken, one from the first cohort and two from the second cohort, as this second cohort was split geographically across health care education sites. The focus group discussions lasted between 42 and 60 min and were audio-recorded [28]. Each group contained 3 to 9 individuals (see Table 2), which is within acceptable parameters for focus groups [28].

Quieter participants were prompted to ensure that each member had the opportunity to contribute to discussions. The stages of the focus groups followed those set out by Ritchie et al. [29], including introducing ourselves and setting ground rules, participant introductions, conducting the discussion, and ending the discussion, with opportunities to add anything else.

### Data analysis

The focus groups ran sequentially and preliminary analysis was undertaken in between the three sessions. By the third focus group, no new categories or themes were emerging and data saturation was therefore reached [29]. Quirkos v1.5.2 software was used to manage the data. Data was analysed thematically using the six steps outlined by Braun and Clarke [27]. Coding and categorising were completed independently by one researcher. A sub-sample was analysed independently by another researcher, then all authors assisted in finalising the themes. This process helps ensure that the quotes provided are not the idiosyncratic views of individuals but are illustrative of points and themes developed across the dataset. The analysis consisted of both semantic (descriptive) and latent (inferential) levels. Descriptive level analysis took the data at face value and reflected it directly in the results; such as those presented in results section 1.1. Latent analysis provided a further level of interpretation such as that noted in results section 3.1 where the tensions and conflict reported can be understood as representing a wider issue of role ambiguity for the TNAs.

### Rigour

Rigour has been enhanced in this study by undertaking systematic and recognised methods of data collection and analysis, providing a detailed description of the study design, and using researcher triangulation [29]. The focus group topic schedule was developed following consultation with 10 key stakeholders involved in TNA commissioning, training and deployment, including NHS managers (*n* = 2), university lecturers (*n* = 2), Health Education England commissioners (*n* = 3) and senior members of the Royal College of Nursing (*n* = 3). Two researchers analysed the transcripts and all authors contributed to the development of themes.

## Results

Three overarching themes were identified following thematic analysis; facilitators of TNA personal growth; factors restricting TNA development; and TNA role ambiguity. Anonymised data extracts have been used to illustrate the themes.

### Facilitators of TNA personal growth

TNAs in this study demonstrated personal growth through affordable career progression by developing new knowledge and embracing wider career opportunities.

#### Affordable career development

Participants had previously worked as healthcare assistants (HCAs) or support workers in diverse fields such as learning disabilities, mental health, surgery, emergency care, orthopaedics, and haematology. Most felt that, prior to undertaking the TNA course, they lacked opportunities for career progression:

*“I’d been working as a support worker for years before doing this course … I wanted to develop a bit more, ‘cause as a Band 3 support worker, there aren’t many opportunities to develop or to move into other things.” (Focus group 1 Scott)*

As unregistered HCAs and support workers, despite extensive experience, participants lacked investment in their roles, both financially and academically. Linked to this, financial responsibilities such as student loans and dependence on a regular income, had previously limited participants from accessing further training:

*“I’ve got a young family so I couldn’t afford to go and get a loan as it is now. I couldn’t even live on a bursary, let alone a loan. So this was the only way of developing for me.” (Focus group 1 Carl)*

For many, especially those with family responsibilities, training close to home was important. It was not convenient to move away to advance their careers, therefore they valued the opportunity for progression within their local hospitals:

*“I think it’s really good that local hospitals … are willing to train their own staff. I think that’s really positive, rather than having to go where the training is, but they’re investing in their own staff, because healthcare assistants, they do want to progress, don’t they, and, a lot of them, felt like they couldn’t.” (Focus group 2 Jane)*

In addition to this desire for career development, some participants viewed the NA role as a mechanism to gain deserved, formal recognition. In this way, the training was important in providing clear justification for increased remuneration:

*“I’m quite happy to admit that finances was a massive thing for me, you know, when you’ve been ten years at the top of your band and you’ve been in a pay freeze in the NHS … I think it’s perfectly understandable when you’ve got a young family to think, I want a bit more money for what I’m doing.” (Focus group 1 Carl)*

TNAs who were well established in their previous roles were not in a position to reduce their income, take on debt, or move locality, so NA training provided an option for progression that many felt they would never experience.

#### New knowledge and opportunities

In addition to the opportunity for career promotion, TNAs also valued the development of new knowledge. They talked about having experienced personal growth through developing new knowledge, skills and opportunities. There was clearly frustration with the constraints of their previous roles and many desired to develop skills to more effectively support registered nurses in improving patient care:

*“I think you sort of get stuck in a bit of a rut when you’ve worked on a ward for so long and then you’re just doing your normal everyday jobs in your little role. I got a bit fed up of just doing my bit and then seeing the nurses struggle and I wanted to be able to do more to support them better, so really excited when this course came up.” (Focus group 2 Julie)*

Several participants reflected on how their increased knowledge led to greater confidence in providing patient care. For example, one TNA explained how she is better placed to provide relevant information to patients and their relatives:

*“If there were phone calls before I’d have to go and find a nurse to discuss with whoever was on the other end of the phone, whereas now I can take responsibility for that call. Or if somebody asks for pain relief, I can look at the drug chart and I can understand what they’ve had, what medication they’ve got left to have. I don’t have to go and find a nurse.” (Focus group 2 Julie)*

In addition to developing new knowledge, TNAs experienced a range of new opportunities during their training, from the wide variety of clinical placements to travelling to conferences.

These experiences not only helped build confidence in delivery of patient care, but also influenced future career aspirations. Some TNAs were content to work in a role that bridged the gap between healthcare assistant and registered nurse, planning to return to their previous workplace.

*“I’m quite happy there. And at the moment, I don’t really have any, sort of … any thoughts of leaving ‘cause I enjoy it. Yeah.” (Focus group 1 Scott)*

Others also planned to continue to work as NAs but in new settings, and some aspired to undertake further training to transition to RNs. Through undertaking a range of placements, TNAs gained insights into a variety of healthcare settings, providing greater possibilities for future career choices:

*“I’ve been to endoscopy and I absolutely loved endoscopy, and I wouldn’t have seen that if I’d just been a HCA on my ward. Whereas district nursing I love that and just going and doing all the different things. It was very interesting. It gives you a wider scope of where you might want to go in the future.” (Focus group 3 Sally)*

Around two-thirds of the TNAs in this study expressed an interest in becoming registered nurses:

*“Yeah, I do want to do my nursing. So as soon as the opportunity comes up I’m probably going to take it, but for the time being … I really want to just get into a job, work for a little bit... So yeah, that’s what I’m thinking.” (Focus group 2 James)*

There was a clear passion for career development among TNA participants, driven by a lack of developmental opportunities in their previous roles and made available through training that was funded and offered locally.

### Factors restricting TNA development

These TNAs were pioneers in their workplaces, lacking role models to emulate or embedded systems of support. Therefore they faced novel challenges relating to their development, such as placement concerns, academic pressures, and unclear career progression.

#### Placement variations and academic pressures

Participants raised several concerns relating to clinical placements, including how they were organised. Those based at a single site, with short ‘alternative’ placements throughout the 2 years described being settled in their role compared to those who moved base placements every 6 months and experienced associated anxiety:

*“It was terrifying moving away from places you’ve been, well, for ages and then to go into a different place, meet new people, then lose those people, off again, start again in six months’ time.” (Focus group 2 James)*

The experience of mentorship and general support during placements was also a concern for some. For example, one TNA identified that her mentors did not have the required qualifications to legitimately support their practice:

*“I was given two mentors but then I got onto the ward and found out that they’ve not actually passed their mentorship course, so I ended up with nobody. For six months I’ve not had anybody.” (Focus group 3 Hannah)*

Others expressed a general feeling of being ‘in the way’ during their clinical placements. Workplaces lacked experience of supporting TNAs, therefore they felt burdened by the task.

*“They all run the other way when they say, “oh will you work with so and so”, they reply “oh no, I’m not doing that!”” (Focus group 3 Kim)*

TNAs experienced variations in supernumerary status and protected learning time during their placements, which, they felt, impacted on their development:

*“Because we’re counted in the numbers, I don’t think we get as much opportunity as we’d like. I think that’s the biggest issue for me.” (Focus group 2 Julie)*

A lack of protected learning time was viewed by some as a barrier to learning. Several participants had limited experience of university and associated academic pressures. Such pressures were highlighted as significant in terms of the level of study but also in terms of the time commitment, alongside their clinical work:

*“You’ll be doing assignments, you’ve got exam revision. And there’s not enough hours. A lot of people naively came into it not expecting that, and I think that’s where a lot of upset was caused: well, how am I going to do this, why am I not having a day to do it.” (Focus group 3 Hannah)*

It is clear that TNAs were enthusiastic about the opportunities to develop their role but faced disappointments in their placement and academic pressures.

#### Unclear career progression

Despite high aspirations to transition to RNs, all participants expressed a lack of clarity about how to access the training. They were unsure whether a transition course would be university-based or distance learning.

*“I asked last week and somebody said that they [tutors] would potentially be writing something while we’re doing this class for a top up. If not it could be like a home learning thing where you stay on your base placement.” (Focus group 3 Anna)*

There was also uncertainty about job opportunities. Participants described competition for jobs and a lack of choice with options governed by areas of high nursing staff shortages:

*“You’re under the impression that basically you can pick where you’re going to go. In reality, the NAs that are due to qualify at [town] have been given jobs in cardio and respiratory where they’re short staffed.” (Focus group 3 Claire)*

It is clearly important for TNAs to have confidence of job security on completion of the course, and clarity around the process of converting their training to becoming registered nurses, however neither are certain.

### TNA role ambiguity

TNAs experienced widespread role ambiguity, both personally and within their organisations. However, in mitigating the adverse effects of this lack of role clarity, they value broad support networks, which functioned as naturally occurring communities of practice.

#### Lack of role clarity

Participants experienced both personal and departmental lack of role clarity. They were often asked to define their role by patients and colleagues.

*“Personally the first question nearly everybody asks you when you see them on placement is “what actually is a TNA?”” (Focus group 1 Scott)*

*“It’s quite stress inducing that though, isn’t it, when you’re trying to explain something you’re not really fully understanding what you’re doing yourself.” (Focus group 1 Carl)*

The inability to explain their role highlights a lack of clear occupational identity. One group explained that the difficulty in describing the role to others was compounded by the late introduction of the NA scope of practice, mid-way through their training [23]. A consequence of this role ambiguity, and perhaps also the lack of supernumerary status, was that TNAs were often expected to work as HCAs during their clinical placements. Their scope of practice sometimes varied throughout the day, and was dependent on the expectations of their managers:

*“On some of the placements, it’s like you’ve been sent to learn how to be a healthcare assistant in another setting.” (Focus group 1 Rob)*

This illustrates the tension between being a ‘trainee’, with associated learning needs, yet also being counted as part of the workforce and expected to deliver care. The lack of experienced NA role models contributed to role ambiguity:

*“You’ve got nobody to follow on from, like I say if you’re … a student nurse, you know what the course is or you know pretty much what it’s going to entail. There’re thousands [of student nurses] everywhere and everybody knows what you’re going to be doing afterwards. For us there isn’t any of that at all.” (Focus group 2 Jane)*

In addition to these tensions in role expectations and lack of role models, TNAs perceived that some RN colleagues felt their jobs were under threat by the emergence of the NA role:

*“Other people have been saying, like, nurses have felt threatened... I think some RNs on wards are maybe seeing us a cheaper replacement.” (Focus group 1 Rob)*

This suggests a lack of consultation and education across organisations regarding workforce changes prior to implementation of the NA role. Participants also found that more experienced RNs compared the NA role to the previous SEN, generating concerns about the potential transience of the NA role:

*“And I think probably more so from like your old school type nurses, older generation, because there was obviously the enrolled nurse, so they like to make sure that it’s not going to be the same as that and it’s just going to phase out again and, well, what’s the point.” (Focus group 2 Jane)*

There was a perception that RNs were reluctant to invest their time and effort in mentoring TNAs if the permanence of the role was in doubt. A lack of role clarity by colleagues was clearly a challenge to TNAs, particularly as they were not entirely sure of their scope of practice themselves. This affected both the supervision and expectation of TNAs, and consequently the experience of TNAs. Despite this role ambiguity and associated role conflict, participants viewed the role as a valuable opportunity for career progression and sought out others to legitimise their position in the healthcare team.

#### Broad support networks

Due to the infancy of the NA role, and subsequent challenges, TNAs relied on broad support networks. These included line managers, academic tutors, and other TNAs (both locally and nationally via social media). Despite some of the problems associated with mentorship, several TNAs received good support from clinical colleagues:

*“I’ve had support from all staff, they’ve been going through this journey with us and they’re in the same boat. They’ve had no clue what’s been happening but they’ve all been accommodating.” (Focus group 2 James)*

This highlights the value of organisational consensus in choosing to make the role a success, striving to facilitate the career development of HCAs, despite widespread ambiguity. In addition to clinical support, several TNAs talked about the support they gained from good relationships with academic tutors.

They valued a TNA ‘community of practice’ in which to share knowledge, experiences and to support each other. This was particularly important considering the lack of qualified NA role models in their workplaces. Although face-to-face support was important to participants, they also gained a wider perspective of other TNAs nationally through a social media group:

*“So we’re part of this Facebook group that’s got all the TNAs in and they were all putting that they were doing all these medications and stuff and we still weren’t allowed to do it.” (Focus group 2 Julie)*

This provided insight into how TNAs across England managed a range of challenges related to the new role, for example, regarding medication administration. It is clear that these TNAs were keen for the role to succeed and be recognised as legitimate members of the healthcare team.

## Discussion

England has introduced a new second-level nursing role, similar to many other high income countries [18]. This study has revealed some of the drivers and challenges faced by student TNAs. TNA personal growth is achieved through affordable local career progression, the acquisition of knowledge and varied workplace opportunities. For some, NA training is seen as the only viable route into nursing in light of the lack of student nurse bursary [14]. The motivation to increase knowledge and skills is consistent with previous studies which identify a lack of career development opportunities for HCAs and support workers [1, 9, 10].

TNAs face a number of challenges including; lack of mentors, academic pressures and unclear career pathways. The inadequate provision of mentors should not be a problem in the future as changes to the NMC standards will ensure that all new nurses and NAs will qualify with the skills to supervise students [23, 31]. The move from ‘supernumerary’ to ‘protected learning time’ time was also raised as an issue by TNAs. This is an inevitable consequence of apprenticeship funding as made clear by the NMC; *“The NMC does not require nursing associate students to be supernumerary while learning in practice, but the student must have protected learning time p2”* [32]. It may be that TNAs need to develop negotiating skills to ensure their learning needs are met. A role evaluation has highlighted the need for TNAs to be assertive when seeking learning experiences [3]. It is important for future studies to explore how ‘protected learning time’ is played out in clinical practice given current contextual pressures.

Furthermore, pathways for career progression were unclear for TNAs. It has been suggested that TNAs could transition to become RNs via an 18-month nursing degree, or two and a half year nursing apprenticeship [19]. However, TNAs in this study were unsure of where and how to apply for such training. It will be important for further research to explore whether this ambiguity around career progression is a widespread problem for TNAs, and whether development opportunities arise.

Some TNAs experienced a lack of clarity concerning their scope of practice relating to drug administration. Due to the late publication of national guidelines for NA practice [23], this was particularly problematic for the pilot cohort who were nearing completion of their course. Role ambiguity is clearly an obstacle for TNAs which is consistent with previous research on the similar assistant practitioner (AP) role. Wakefield et al. [9] identified role ambiguity as a consequence of the lack of regulation and clarity of scope of practice in the AP role. APs have been described as sitting between two occupational spaces; as neither professional staff, due to the lack of regulation, nor support staff, due to the extra responsibilities expected [9]. Although TNAs also blur boundaries with nursing, they differ from APs as they are regulated with clear standards of practice developed by the Nursing and Midwifery Council, the national professional body for nurses [23].

Some TNAs perceived that their nurse colleagues felt threatened by the new NA role. Similarly, Traynor et al. [6] found that the AP role challenged the unique skills previously owned by nursing. The Nursing and Midwifery Council outlines that NAs can undertake many traditional nursing tasks, therefore blurring the boundaries between the roles, but cannot make autonomous decisions about care planning [23]. Traynor et al. [6] argue that nursing has retained professional power over APs through a hierarchy of organisational accountability. This is achieved, firstly, by the aspiration of APs to becoming RNs and secondly by the configuration of paraprofessional groups, largely trained by nurses. In the present study, TNAs are similarly trained by nurses, and most aspire to transition to becoming RNs. Therefore, it might be argued that they should not pose a threat to the professional identity of nursing [6].

TNAs made use of a range of social networks (face-to-face and via social media) forming a community of practice to mitigate the consequences of role ambiguity, and other factors that hindered their development, inevitable in the implementation of new roles [33]. Previous new roles in healthcare have benefitted from forming communities of practice, which have been found useful in identity building [4, 25].

It will be important for future research to explore the impact of academic preparedness and pressures raised in this study and to evaluate the clinical impact of TNAs in terms of inter-professional working, patient satisfaction, and adverse events. The impact on the skill mix change should also be explored.

### Limitations

Data collection was limited to three focus groups of TNAs based at one University. TNAs all had backgrounds working as HCAs which may not represent TNAs of the future. Future studies, including the planned longitudinal cohort study, should therefore explore experiences of TNAs from a wider range of organisations and occupational backgrounds.

## Conclusions

Affordable local career progression is an important driver for undertaking NA training. TNAs face placement and academic challenges in addition to role ambiguity, finding face-to-face and online support networks crucial to managing these issues.

The growth in numbers of nursing associates (NAs) will undoubtedly impact on the very similar, but unregistered assistant practitioner (AP) role, leading to role confusion. Policy makers should be aware of the factors that promote and hinder TNA development when considering recruitment and retention strategies. Educators and employers should ensure role clarity to improve occupational identity for TNAs, patients and wider healthcare teams. The results from this study will contribute to the global evidence relating to the development of new nursing roles under development in similar healthcare systems.

## Data Availability

The datasets used and/or analysed during the current study are available from the corresponding author on reasonable request.

## Abbreviations

AP: Assistant Practitioner
HCA: Healthcare assistant
NA: Nursing Associate
NMC: Nursing and Midwifery Council
RN: Registered Nurse
SEN: State Enrolled Nurse
TNA: Trainee Nursing Associate
